# An Account of Diseases in an Eastern District of London, from Aug. 20 to Sept. 20, 1810

**Published:** 1810-10

**Authors:** 


### *An Acconnt of Diseases in an Eastern District of London, from Aug. 20 to Sept. 20, 1810.*

| | |
|---|---|
| **ACUTE DISEASES.** | |
| Scarlatina | 3 |
| Scarlatina Anginosa | 7 |
| Variola | 3 |
| Rubeola | 4 |
| Rheumatismus Acutus | 2 |
| **CHRONIC DISEASES.** | |
| Catarrhus | 7 |
| Tussis | 10 |
| Dyspnœa | 5 |
| Tussis cum Dyspnœa | 9 |
| Pleurodyne | 4 |
| Vertigo | 5 |
| Paralysis | 2 |
| Cephalalgia | 7 |
| Amenorrhœa | 6 |
| Fluor Albus | 7 |
| Dysuria | 3 |
| Obstipatio | 4 |
| Enterodynia | 6 |
| Rheumatismus Chronicus | 10 |
| **PUERPERAL DISEASES.** | |
| Abscessus Mammæ | 1 |
| Menorrhagia Lochialis | 2 |
| Dolores post Partum | 5 |
| Dysuria | 4 |
| **INFANTILE DISEASES.** | |
| Pertussis | 6 |
| Scrophula | 4 |
| Herpes | 3 |
| Aphthæ | 4 |

During

During the last few weeks, several instances of scarlatina have occurred. In these cases the scarlet efflorescence extended over the whole surface of the body, and was attended with a considerable degree of fever. The pulse has in general been small and irregular, and symptoms of languor and depression have been observed throughout the disease. The head has sometimes been considerably affected with pain, and in one of the cases there was a slight delirium. In addition to these symptons, in some instances, there were very painful affections of the throat, accompanied with aphthous appearances, and sometimes with deep ulcerations in the tonsils; thus constituting the disease which Nosologists have denominated Scarlatina Anginosa. At the commencement of this disease, though bleeding is seldom admissible, we shall generally derive advantage from a cooling diet and regimen, together with the use of saline febrifuge medicines. As the disease advances, and if the surface changes its appearance from a florid red to a mark and livid colour, and the throat begins to wear a more unfavourable aspect, the use of bark and acids, of wine and cordials, will become necessary to support the strength of the patient, and to conduct to a favourable termination.

---

*Since the First Page of this Number was printed, an arrangement has been made with Dr. SAMUEL FOTHERGILL, to conduct this Work in future, as far as regards the Practice of Medicine; and with WILLIAM ROYSTON, Esq. for the Department of Surgery. Such a change has long been called for by the Public, and the Proprietor considers himself fortunate in having effected it in a way which augurs so favourably to the future credit of the work.*

*Communications are requested to be addressed as heretofore to the care of Sir Richard Phillips, the Proprietor, at No. 6, Bridge-Street, London.*

